# Significance of Circulating Cell-Free DNA Species in Non-Alcoholic Fatty Liver Disease

**DOI:** 10.3390/ijms22168849

**Published:** 2021-08-17

**Authors:** Lampros Chrysavgis, Alkistis Papatheodoridi, Evangelos Cholongitas, Michael Koutsilieris, George Papatheodoridis, Antonios Chatzigeorgiou

**Affiliations:** 1 Department of Physiology, Medical School of National and Kapodistrian University of Athens, 11527 Athens, Greece; lchrisaugis@gmail.com (L.C.); alkistispapath@gmail.com (A.P.); mkoutsil@med.uoa.gr (M.K.); 2 Department of Clinical Therapeutics, “Alexandra” General Hospital of Athens, Medical School of National and Kapodistrian University of Athens, 11528 Athens, Greece; 3 1st Department of Internal Medicine, General Hospital of Athens “Laiko”, Medical School of National and Kapodistrian University of Athens, 11527 Athens, Greece; cholongitas@yahoo.gr; 4 Department of Gastroenterology, General Hospital of Athens “Laiko”, Medical School of National and Kapodistrian University of Athens, 11527 Athens, Greece; gepapath@med.uoa.gr; 5 Institute for Clinical Chemistry and Laboratory Medicine, University Clinic Carl Gustav Carus, Technische Universität Dresden, 01307 Dresden, Germany

**Keywords:** non-alcoholic fatty liver disease (NAFLD), cell-free DNA (cf-DNA), genomic DNA, liver cirrhosis, liquid biopsy

## Abstract

The pathogenetic mechanisms involved in the progression of non-alcoholic fatty liver disease (NAFLD) have not been completely elucidated, while the significance of circulating cell-free DNA (cf-DNA) species has been rarely evaluated in NAFLD. Herein, we assessed the serum levels of cf-DNA species in NAFLD patients and investigated their potential associations with patients’ characteristics and severity of liver disease. Forty-nine adult patients with NAFLD of any stage were included in this cohort study. Cf-DNA was isolated from patients’ sera and the levels of several distinct cf-DNA species including total cf-DNA, gene-coding cf-DNA, Alu repeat sequences, mitochondrial DNA copies and 5-methyl-2′-deoxycytidine were determined. Cirrhotic compared to non-cirrhotic patients had significantly lower serum levels of cf-DNA and RNAse P coding DNA as well as higher expression of 5-methyl-2′-deoxycytidine. After adjustment for the significant clinico-epidemiological factors, lower serum levels of cf-DNA or RNAse P were independently associated with the presence of cirrhosis. Serum levels of total and gene-coding DNA are associated with the presence of cirrhosis in NAFLD patients regardless of clinical or epidemiological parameters and may therefore be used as a screening tool for NAFLD progression.

## 1. Introduction

The global prevalence of non-alcoholic fatty liver disease (NAFLD) is estimated to be approximately as high as 24% [1] increasing along with the increasing prevalence of metabolic comorbidities such as metabolic syndrome, insulin resistance or type 2 diabetes mellitus (T2DM), obesity and atherogenic dyslipidemia [2]. NAFLD is the second most common cause for liver transplantation in USA and will probably emerge as the leading cause of end-stage liver disease in the near future [3]. NAFLD encompasses a wide spectrum of histological lesions ranging from simple steatosis or non-alcoholic fatty liver (NAFL) to non-alcoholic steatohepatitis (NASH) [2,4,5] associated with development of fibrosis of variable severity including cirrhosis and even hepatocellular carcinoma (HCC) in up to 10–15% of cases [2,6]. However, the pathogenetic mechanisms involved in the development and progression of NAFLD and NASH have not been completely elucidated.

Circulating cell-free DNA (cf-DNA) consists of small nucleic acid fragments that are released from lysed or apoptotic cells into the blood stream. Though, the pool of serum cf-DNA, as an entity, encompasses beyond the circulating fragments of nuclear gene-coding and non-coding DNA, the Alu repeat sequences of cf-DNA and mitochondrial DNA (mitoDNA), collectively referred to as DNA species [7]. Previous studies have assessed the implication of cf-DNA as a surrogate marker for risk stratification and management of cancer patients [8,9] and its involvement as diagnostic biomarker in inflammatory liver disease [10]. In addition, epigenetic alterations, predominantly those reflected by DNA methylation have been associated with a plethora of human diseases ranging from neurological and psychiatric disorders to tumorigenesis [11]. Nonetheless, only few studies have extensively focused on the potential significance of cf-DNA for the progression of NAFLD, while information pertinent to the importance of the distinct circulating cf-DNA species to this direction remains scarce [12,13].

Therefore, this study aimed to determine the serum levels of several cf-DNA species and epigenetic alterations in patients with NAFLD and to evaluate their potential associations with patients’ characteristics and severity of liver disease.

## 2. Results

The main clinical characteristics of 49 patients are presented in Table 1. Cirrhotic compared to non-cirrhotic patients were older (*p* < 0.001), had increased prevalence of T2DM, increased fasting glucose levels (*p* < 0.050) as well as significantly lower serum albumin levels and platelet counts and increased international normalized ratio (INR) (all *p* < 0.001). Demographic and anthropometric parameters including gender, ethnicity, BMI, waist and neck circumference, alcohol consumption and smoking habits as well as fasting insulin did not differ significantly between the two groups.

### 2.1. Circulating cf-DNA and DNA Methylation Markers in Relation to NAFLD Severity

**Figure 1:**
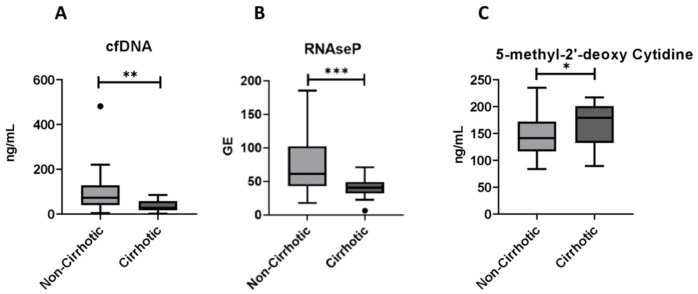
Serum levels of cell-free DNA (cf-DNA, ng/mL) (**A**), RNAse P coding DNA (GE: genomic equivalents) (**B**) and 5-methyl-2′-deoxycytidine (ng/mL) (**C**) in non-cirrhotic and cirrhotic patients with non-alcoholic fatty liver disease. Data are presented in whisker plots. Whisker plots show median values and 25–75th percentiles. * *p <* 0.05, ** *p <* 0.01, *** *p <* 0.001.

The circulating levels of cf-DNA markers in relation to the severity of NAFLD are presented in Table 2. Cf-DNA concentration was significantly lower in cirrhotic compared to the non-cirrhotic patients (72.6 vs. 27.9 ng/mL, *p* = 0.002) (Figure 1A). Consistent with that, the levels of coding genomic DNA, as assessed by RNAse P gene quantification were significantly lower in cirrhotic patients too (70.2 vs. 39.9 GEs, *p* < 0.001) (Figure 1B). Nonetheless, the mean levels of Alu115, Alu247 as well as Alu247/Alu115 ratio as an index of DNA integrity did not differ significantly between the cirrhotic and non-cirrhotic cases. Along this line, the mean mitochondrial DNA copy number was also similar between those two groups. The mean levels of 5-methyl-2′-deoxycytidine as a surrogate marker of DNA methylation were significantly higher in the cirrhotic than non-cirrhotic patients (170.3 vs. 145.1 ng/mL, *p* = 0.029) (Figure 1C). In the non-cirrhotic group, there was no significance difference between NAFL and NASH cases in the serum levels of the distinct cf-DNA species, namely total cf-DNA (72.9 vs. 68.4 ng/mL, *p* = 0.773), RNAse P coding DNA (83.6 vs. 63.8 GEs, *p* = 0.214), Alu115 (131.4 vs. 104.8 GEs, *p* = 0.276), Alu247 (39.9 vs. 31.1 GEs, *p* = 0.265), Alu247/115 ratio (0.29 vs. 0.30, *p* = 0.667) as well as in DNA methylation (144.2 vs. 145.6 ng/mL, *p* = 0.919). There was only a trend towards higher mitochondrial DNA copies in NASH compared to NAFL cases (19 vs.26 copies, *p* = 0.088).

### 2.2. Associations between Circulating cf-DNA and DNA Methylation Markers with Other Patient Characteristics, Laboratory Parameters and Histological Findings

**Figure 2:**
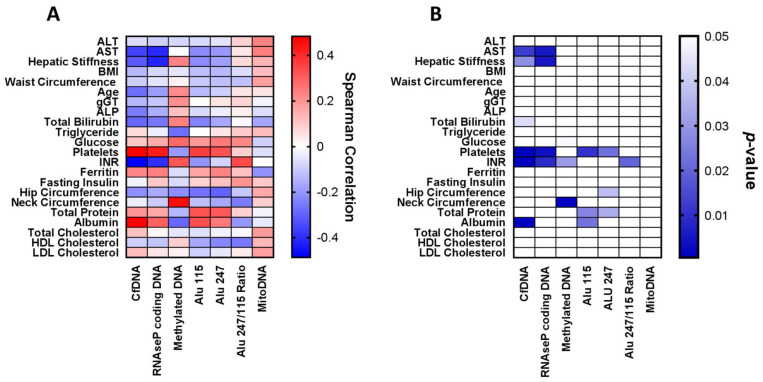
Heatmaps showing correlations between serum levels of cell-free DNA (cf-DNA), RNAse P coding DNA, methylated DNA assessed as levels of 5-methyl-2′-deoxycytidine, Alu115, Alu247, Alu247/Alu115 ratio and mitochondrial (mito) DNA with anthropometric and laboratory parameters of patients with non-alcoholic fatty liver disease. Heatmap (**A**) shows Spearman’s correlation coefficients according to their ranks and heatmap (**B**) shows their respective *p*-values (*p*-values > 0.05 are shown as white cells).

Significantly robust associations between DNA species and clinical, anthropometric or biochemical patients’ characteristics were observed (Figure 2).

**Figure 3:**
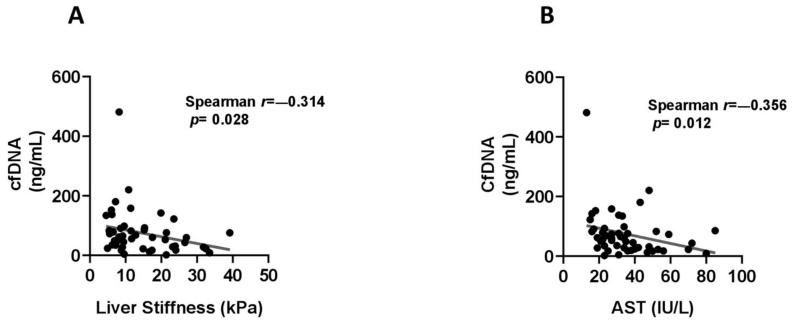
Correlations between serum levels of cell-free DNA (cf-DNA, ng/mL) with liver stiffness (**A**) and aspartate aminotransferase (AST) levels (**B**) using Spearman’s rank correlation coefficients.

Notably, cf-DNA was correlated inversely to liver stiffness (r = −0.314, *p* = 0.028) (Figure 3A), AST levels (r = −0.356, *p* = 0.012) (Figure 3B) and INR (r = −0.486, *p* = 0.001) and positively to platelet counts (r = 0.477, *p* = 0.001) and albumin levels (r = 0.483, *p* = 0.001).

**Figure 4:**
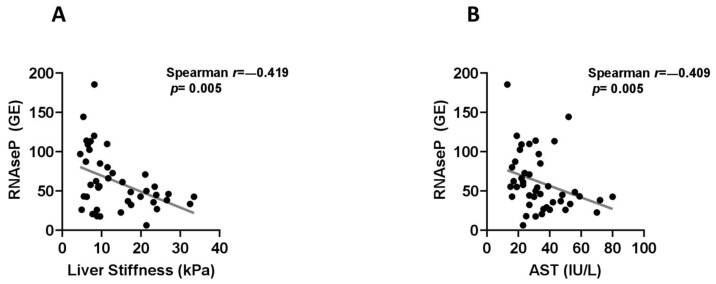
Correlations between serum levels of RNAse P coding DNA (GE: genomic equivalents) with liver stiffness (**A**) and aspartate aminotransferase (AST) levels (**B**) using Spearman’s rank correlation coefficients.

Along this line, serum levels of the RNAse P gene were found to have similar correlations to liver stiffness (r = −0.419, *p* = 0.005) (Figure 4A), AST levels (r = −0.409, *p* = 0.005) (Figure 4B), INR (r = −0.398, *p* = 0.009) and platelet counts (r = 0.426, *p* = 0.004). The mean levels of Alu115 were positively correlated to total protein (r = 0.328, *p* = 0.026) and albumin serum levels (r = 0.335, *p* = 0.023) as well as to platelet counts (r = 0.363, *p* = 0.010), while similar positive associations were observed between Alu247 levels with total protein (r = 0.314, *p* = 0.034) and platelet counts (r = 0.326, *p* = 0.022). Finally, the Alu247Alu/115 ratio had a positive correlation with INR (r = 0.342, *p* = 0.020) (Figure 2). Furthermore, RNAse P coding DNA levels were inversely correlated with fibrosis stage (r = −0.470, *p* = 0.024). Nonetheless, we did not distinguish any remarkable correlation between the remaining cf-DNA parameters with steatosis, inflammation or ballooning grades except for a tendency for inverse correlation between ballooning grade and RNAse P gene (r = −0.370, *p* = 0.083) and a trend for positive correlation between ballooning grade and copies of mitoDNA (r = 0.416, *p* = 0.077).

### 2.3. Independent Associations of cf-DNA Parameters with Presence of Cirrhosis

**Figure 5:**
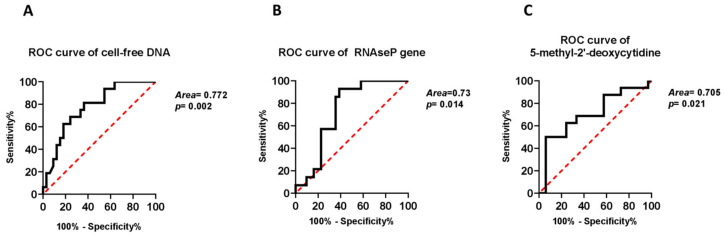
Area under receiving operating characteristic (ROC) curve of cf-DNA (**A**), RNAse P coding DNA (**B**) and 5-methyl-2′deoxycytidine (**C**) serum levels for the detection of NASH-related liver cirrhosis in NAFLD patients.

Next, we sought to determine whether the differentially expressed cf-DNA species measurements performed in our study, along with clinicο-epidemiological parameters could be associated with the development of cirrhosis in our cohort of patients. The results of the multivariable analyses models are presented in Table 3. Elevated levels of circulating cf-DNA were significantly associated with decreased probability of cirrhosis after adjustment for age and presence of T2DM (OR: 0.974, 95% CI: 0.951-0.997; *p* = 0.026). Similarly, increased serum levels of amplifiable genomic DNA were markedly related to decreased odds for cirrhosis in a model adjusted for the same parameters (OR: 0.945, 95% CI: 0.896-0.996; *p* = 0.035). In contrast, the serum expression of 5-methyl-2′-deoxycytidine, as a surrogate marker of total cf-DNA methylation, was not found to represent a factor independently associated with presence of cirrhosis (OR: 1.014, 95% CI: 0.994–1.035; *p* = 0.163). Next, we sought to determine whether the cf-DNA species measurements, performed in our study, could be implemented so as to detect the presence of liver cirrhosis in our cohort of patients using ROC curves. Indeed, the area under ROC curve showed that the serum levels of both cf-DNA and RNAse P coding DNA provided sufficient discrimination of cirrhosis (AUC: 77.2%, *p* = 0.002 and AUC: 73%, *p* = 0.014, respectively) (Figure 5A,B). Consistently, serum cf-DNA methylation, as evaluated by the circulating levels of 5-methyl-2′-deoxycytidine could be also considered a valid marker of existence of cirrhosis (AUC: 70.5%, *p* = 0.021) (Figure 5C).

## 3. Discussion

In light of the rising global burden of NAFLD, the better understanding of the pathogenesis of liver disease progression and particularly of development of cirrhosis is of clinical interest. The development of NASH and liver cirrhosis is considered to be mediated by inflammatory and DNA damage response-associated mechanisms which drive the hepatocytes to apoptosis and necrosis [14,15,16]. During this long-term process, DNA as well as other intracellular components such as nucleosomes [17] or micro-RNAs [18] are released into the bloodstream. Cf-DNA has recently been reported as a biomarker for risk stratification and prediction in a variety of human disorders including cancer and inflammatory liver diseases [9], while a previous genome-wide study demonstrated that liver constitutes a major source of the cf-DNA pool that circulates freely in the plasma [19].

In our study, we evaluated the associations of total cf-DNA, comprised of gene-coding, non-coding and repeated sequences as well as mitochondrial DNA copy number with the progression of NAFLD and particularly with the presence of cirrhosis. Of interest, serum levels of both cf-DNA and RNAse P, as an indicator of amplifiable genomic DNA [20], were significantly lower in cirrhotic than non-cirrhotic patients with NAFLD regardless of important clinicο-epidemiological factors such as age and presence of T2DM. In fact, in multivariable models including age and presence of T2DM, a decrease of amplifiable serum levels of total cf-DNA by 10 ng/mL increased the odds for cirrhosis by 23% and a corresponding reduction of RNAse P serum levels by 10 GEs led to 43% higher odds for presence of cirrhosis. Along this line, significant inverse correlations were also shown between liver stiffness and AST with serum levels of both cf-DNA and RNAse P, while a significant inverse correlation between RNAse P and liver fibrosis stage was also observed. In a previous study, Karlas et al. demonstrated a positive correlation between liver stiffness and concentration of small fragments of cf-DNA [21]. However, in this study, only non-invasive methods were used for the characterization of NAFLD severity whereas in our study, the gold standard of liver biopsy was predominantly used for the diagnosis and staging of NAFLD. Thereafter, we evaluated the diagnostic value of each of those biomarkers using area under ROC curve analysis. Serum expression of cf-DNA, RNAse P coding DNA and 5-methyl-2′-deoxycytidine displayed reliable discrimination of NASH-related cirrhosis reaching area under the ROC as high as 77.2%, 73% and 70.5%, respectively. In the course of NAFLD and its progression to NASH, the hepatocytes undergo cell death, necrosis or cellular senescence while the regenerative liver mechanisms are activated to restore the damaged liver tissue [14]. During this process, the apoptotic or senescent cells release DNA into the circulation. Eventually, the transition to cirrhotic state is a terminal point for liver regeneration capacity [22], during which the deposition of extracellular collagen matrix robustly impedes the restoration of the normal liver structure, while the architecture of the hepatic parenchyma faces permanent damage. This likely results in decreased cf-DNA release into the circulation from hepatocytes and their neighboring cells [23]. Given that cf-DNA has short half-life and provides an instant reflection of disease activity [8] and since cirrhosis represents an established condition, the reduced levels of cf-DNA observed in the group of cirrhotic patients might be attributed to the overall suppression of the disease activity in this group as compared to the non-cirrhotic patients.

We did not find any significant difference in the levels of the remaining cf-DNA species, namely Alu repeats and mitochondrial DNA, between cirrhotic and non-cirrhotic patients of our cohort. The utility of Alu repeats, Alu247/Alu115 ratio and mitochondrial cf-DNA levels as biomarkers was previously proposed predominantly for malignancies including HCC, rather than chronic diseases [24,25]. Thus, the fact that we did not observe any remarkable alteration in the levels of these types of cf-DNA between cirrhotic and non-cirrhotic patients might be attributed to the exclusion of patients with HCC or any other malignancy in our study.

The increased levels of 5-methyl-2′-deoxycytidine in the group of cirrhotic patients as compared to the non-cirrhotic ones, indicating greater DNA methylation was another interesting finding of our study. According to previous reports, the differential methylation of peroxisome proliferator-activated receptor γ within the pool of cf-DNA could serve as a surrogate marker for stratification of fibrosis severity, suggesting that liver DNA methylation changes could be reflected in the blood [26]. Moreover, it has been previously shown that NAFLD progression is associated with hypermethylation and subsequent downregulation of the expression of genes involved in metabolic processes accompanied with hypomethylation and upregulation of genes implicated in liver regeneration and tissue repair [27]. Such data are consistent with our finding that cirrhotic patients displayed increased global DNA methylation levels, presumably associated to reduced expression of genes associated with liver lipid, retinoid and glycolytic liver metabolism as compared to the patients suffering from NAFL or NASH. It should be noted, however, that serum levels of 5-methyl-2′-deoxycytidine were not found to be associated with presence of cirrhosis after adjustment for age and T2DM.

Interestingly, in the non-cirrhotic patients, there was no significant difference in the levels of circulating cf-DNA species between patients with NAFL and NASH, suggesting that presence of cirrhosis is the determinant factor affecting the serum abundance of cf-DNA species. However, we cannot exclude a type II error due to the relatively small sample size of these two groups, and thus statistical power might have not been adequate to reveal such potential differences in circulating levels of cf-DNA species.

Our study is the first trying to assess the significant implication of circulating cf-DNA species within the range of NAFLD, from NAFL to liver cirrhosis. All patients were enrolled during 2019 and early 2020, so our sample collection was relatively recent. Specimens were aliquoted and stored at −80 °C, thus we avoided any variability or degradation that may be caused by long-term freezing storage and repeated thawing–freezing cycles which is known to affect the yield and purity of DNA-containing samples [28]. On the other hand, the main limitation of our study was the relatively limited number of patients which may have affected the statistical power of our analyses.

In summary, our findings suggest the importance of the serum levels of total cf-DNA and gene-coding DNA and potentially of cf-DNA methylation status during NAFLD pathogenesis and particularly in the presence of cirrhosis. Total and genomic cf-DNA were independently associated with the cirrhotic status in multivariable analyses adjusted for significant clinical and epidemiological factors. Thus, further extended studies are needed to shed more light into the validity of the circulating cf-DNA parameters as a liquid biopsy screening tool during NAFLD, its progression to cirrhosis and ultimately the development of HCC.

## 4. Materials and Methods

### 4.1. Patients

Forty-nine consecutive patients with NAFLD of variable severity, ranging from simple steatosis to NASH and even cirrhosis were included in this study. All patients were followed at the liver clinics of the Academic Gastroenterology Department of General Hospital of Athens “Laiko” and fulfilled the following inclusion criteria: (a) confirmed NAFLD diagnosis, (b) age between 18 and 80 years at the time of NAFLD diagnosis, (c) available liver biopsy or diagnosis of cirrhosis based on widely accepted criteria, (d) available stored serum sample, (e) willingness to provide written informed consent to participate in the study. Patients with decompensated cirrhosis, HCC or any other malignancy and/or other factors of liver injury were excluded.

This study was conducted according to the Declaration of Helsinki and was approved by the Ethics Committee of the National and Kapodistrian University of Athens. In addition, a written informed consent for anonymous use of their data and biological material was obtained from all patients participating in this study.

### 4.2. NAFLD Diagnosis and Staging

NAFLD was initially diagnosed in patients with elevated alanine aminotransferase (ALT) and gamma-glutamyl transferase (GGT) and ultrasonographic evidence of hepatic steatosis after exclusion of other causes of liver injury. Regarding the exclusion of other causes of liver injury, all patients were negative for HBsAg, anti-HCV and anti-HIV, did not consume excessive amount of alcohol defined as >210 gweek for males and >140 g/week for females, did not receive any steatogenic medication (corticosteroids, amiodarone, tamoxifen, etc.) or any other potential hepatotoxic agent, did not have autoimmune hepatitis, Wilson’s disease of hemochromatosis and did not suffer from any systematic disease possibly affecting hepatic parenchyma.

In all patients without cirrhosis, the diagnosis of NAFLD was based on standard histological findings [29] and exclusion of other causes of liver injury. The classification of non-cirrhotic patients into cases with NAFL and NASH was also based on standard histological criteria [29]. The diagnosis of NAFLD-related cirrhosis was based on standard histological findings [29] and exclusion of other causes of liver injury in some patients as well as in presence of ≥2 features indicative of metabolic syndrome, exclusion of other causes of liver injury and radiological findings of cirrhosis in other patients. Features indicative of metabolic syndrome included (a) increased body mass index (BMI ≥25 kg/m^2^) or waist circumference (male/female ≥94 cm/80 cm), (b) presence of T2DM, (c) dyslipidaemia (fasting triglycerides ≥150 mg/dL or fasting high density lipoprotein (cholesterol) <40/50 mg/dL in males/females or on treatment for dyslipidemia), (d) hypertension (systolic or diastolic blood pressure ≥130 or ≥85 mmHg, or on treatment for hypertension). Radiological findings of cirrhosis included nodules in the hepatic parenchyma, spleen >12 cm or portal vein >16 mm at abdominal ultrasonography or computed or magnetic resonance tomography or liver stiffness measurement >14 kPa at reliable liver elastography.

Based on the above criteria, 10 patients were diagnosed to have NAFL and 23 patients were diagnosed to have NASH without cirrhosis, whereas NASH-related cirrhosis was diagnosed in 16 of the 49 patients. The diagnosis of cirrhosis was based on histological findings from liver biopsies in 9 of the 16 cirrhotic patients.

### 4.3. Demographic and Laboratory Data

Demographic data such as age, gender, weight, height, alcohol consumption, smoking habits, chronic and occasional medication and biochemical parameters including liver function tests namely ALT, aspartate aminotransferase (AST), γ-GT, alkaline phosphatase (ALP), glucose tolerance markers such as fasting glucose and insulin and relevant data were determined at the day of liver biopsy or the baseline visit for patients with cirrhosis diagnosed without a liver biopsy.

A blood sample from each patient was collected on the day of determination of biochemical parameters. It was then centrifuged at 1500× *g* for 15 min and the serum layer was carefully transferred to a new vial and stored at −80 °C for further use.

### 4.4. Circulating cf-DNA Species and DNA Methylation Markers

After equilibration of the stored serum samples initially at 4 °C and thereafter to room temperature, an additional centrifugation of the samples at 400× *g* for 2 min in order to remove any possible remaining cell components was performed. Cf-DNA was extracted from 200 μL serum using the “Plasma/Serum Cell-Free Circulating DNA Purification Mini Kit” from Norgen Biotek (Thorold, ON, Canada) and stored at −80 °C until further use.

The concentration of the serum cf-DNA was quantified by quantitative real time PCR (qPCR) using the BIORAD iQ5 PCR cycler. Specifically, the housekeeping gene glyceraldehyde 3-phosphate dehydrogenase (GAPDH) with the following primers was used: GAPDH-F: 5′-GGAAGGTGAAGGTCGGAGTC-3 and GAPDH-R: 5′-GAAGATGGTGATGGGATTTC-3. All samples were analyzed in duplicates and the mean concentration of the two measurements was used as the final value. To obtain the exact cf-DNA concentration, a standard curve was generated using (human) “TaqMan Control Genomic DNA” (Applied Biosystems^TM^ Cat No. 4312660, Waltham, MA USA).

RNAse P gene is a single copy gene encoding the RNA subunit of ribonuclease P enzyme required for the site-specific cleavage of the 5′ leader sequence of precursor tRNAs. The serum expression of the RNAse P gene which serves as a marker of serum gene-coding cf-DNA, was evaluated by using the “TaqMan^TM^ RNAseP Detection Reagnets Kit” (Applied Biosystems^TM^ Cat No. 4316831, Waltham, MA USA). This value was expressed on genomic equivalents (GE), where 1 GE corresponds to 3.3 picograms of human genomic DNA [30].

The assessment of the GEs of Alu115 and Alu247 repeat sequences within the cf-DNA was determined by qPCR using the following primers; Alu115-F: 5-GGAGGCTGAGGCAGGAGAA-3, Alu115-R: 5-ATCTCGGCTCACTGCAACCT-3 and Alu247-F: 5-CAAGACTTAGTACCTGAAGGGTGAA-3 and Alu247-R: 5-CTTGCCTCTTTCCTAGCACTG-3. For both the quantification of RNAse P gene and Alu sequences concentrations, facsimile standard curves were constructed using the “TaqMan Control Genomic DNA” from Applied Biosystems as above.

Furthermore, the copies of mitochondrial cf-DNA were evaluated by the use of “Human Mitochondrial DNA (mtDNA) Monitoring Primer Set” (Takara Bio Cat No. 7246,Shiga, Japan) following the kit’s instructions.

Real time qPCR was performed using the “Kapa SYBR Fast qPCR Master Mix (2X) Kit” (Kapa Biosystem Cat No. KK 4608, Cape Town, South Africa as master mix for all assays, except for the amplification of the RNAse P gene, for which the “LUNA Universal Probe qPCR Master Mix” (New England Biolabs Inc. Cat No. M3004,Ipswich, MA, USA) was used.

For all the aforementioned qPCR assays run by the BIORAD iQ5, the cycling conditions were as follows: initial activation for 3 min at 95 °C and 50 cycles of denaturation at 95 °C for 3 s followed by annealing and extension at 60 °C for 30 s. The amplification reaction was followed by melting point analysis at 65–95 °C. Non-template controls were included on each PCR plate to confirm absence of contamination.

The circulating levels of 5-methyl-2′-deoxycitidine, as an indicator of DNA methylation, were quantified by utilizing serum samples and the “DNA Methylation Elisa Kit” (Cayman Chemical Cat No. 589324, Ann Arbor, MI, USA)following the manufacturer’s instructions.

### 4.5. Statistical Analysis

All statistical analyses and graphs were performed with the use of Graph Pad Prism (GraphPad Software, Inc., San Diego, CA, USA) or the SPSS software (IBM Company, Chicago, IL, USA). Kolmogorov–Smirnoff test was used to assess the distribution of each quantitative variable and then the latter were analyzed by unpaired Student’s *t*-test and Mann–Whitney U test as appropriate. Normally distributed quantitative variables were presented as mean values (standard deviation), while non-normally distributed quantitative variables were presented as median values {interquartile range}. Correlations between quantitative variables were assessed by Spearman’s correlation and expressed by Spearman coefficient (r), utilizing data for the indicated parameters as available as possible. The ROUT method for identification of outliers was performed for the analyses of each quantitative variable and during RNase P gene analyses two outliers were detected and subsequently removed. Corrected Chi-squared or two-sided Fisher’s exact test was used for comparison between two categorical variables. Multivariable logistic regression analyses were used in order to identify the potential independent associations of cf-DNA parameters with presence of cirrhosis. Each significantly associated cf-DNA parameter was included in model of multivariable analyses together with the clinico-epidemiological patients’ characteristics associated with cirrhosis in the univariable analyses at a significance level of *p*-value < 0.10. Adjusted odds ratios (OR) and their 95% confidence intervals (CI) are reported. In addition, the performance of significant cf-DNA species as independent markers of NASH-related cirrhosis were evaluated by the area under receiver operating characteristic (ROC) curve and the corresponding confidence intervals were calculated by Wilson/Brown method. Statistical significance for all tests was defined as *p*-value < 0.05.

## Figures and Tables

**Table 1:** Main characteristics of 49 patients with non-alcoholic fatty liver disease in relation to their disease severity: non-alcoholic fatty liver (NAFL), non-alcoholic steatohepatitis (NASH) and cirrhosis.

	NAFL (*n* = 10)	NASH (*n* = 23)	No Cirrhosis ^1^ (NAFL+NASH)	Cirrhosis ^2^ (*n* = 16)	*p*-Value (1 vs. 2)
Age, years	52.9 (8.6)	55.6 (11.1)	54.7 (10.4)	68.1 (8.6)	<0.001
Sex, males (%)	7 (70)	10 (43.5)	17 (51.5)	9 (56.3)	0.755
BMI, kg/m^2^	28.3 (3.1)	32.6 (5.4)	31.3 (5.2)	32.0 (5.2)	0.649
WC, cm	103 (5)	111 (11)	108 (10)	113 (13)	0.176
Alcohol, *n* (%) No use Mild use	8 (80) 2 (20)	20 (90.9) 2 (9.1)	28 (87.5) 4 (12.5)	14 (93.3) 1 (6.7)	0.545
Smoking, *n* (%) Never Former Current	5 (50) 2 (20) 3 (30)	7 (30.4) 8 (34.8) 8 (34.8)	12 (36.4) 10 (30.3) 11 (33.3)	7 (43.8) 6 (37.5) 3 (18.8)	0.570
T2DM, *n* (%)	3 (30)	13 (59.1)	16 (50)	13 (81.3)	0.037
LSM, kPa	6.7 1.6}	9.6 {4.7}	8.8 {4.9}	22.3 {10}	<0.001
ALT, IU/L	34 {30}	35 {34}	35 {37}	32 {28}	0.394
AST, IU/L	22.5 {13}	31 {15}	27 {15}	47 {39}	0.002
ALP, IU/L	86 {64}	72 {38}	73 {43}	123 {53}	<0.001
GGT, IU/L	132 {136}	30 {43}	51 {150}	100 {214}	0.018
Albumin, g/L	46 {5}	47 {4}	46 {4}	36 {9}	<0.001
Bilirubin, mg/dl	1.0 {1.0}	0.5 {0.5}	0.7 {0.7}	1.0 {0.8}	0.013
Ferritin, mg/dl	230 {238}	105 {198}	130 {186}	50 {99}	0.030
Platelets, ×10^9^/L	226 (58)	254 (81)	245 (75)	134 (64)	<0.001
INR	0.98 (0.06)	1.01 (0.08)	1.00 (0.07)	1.29 (0.45)	0.030
Glucose, mg/dl	104 (20)	107 (17)	106 (18)	141 (54)	0.020
Insulin, pmol/L	17 {34}	18 {9}	18 {13}	23 {16}	0.272
LDL, mg/dl	95 (36)	106 (42)	103 (40)	80 (21)	0.055
HDL, mg/dl	54 (22)	54 (14)	54 (16)	50 (16)	0.447
TG, mg/dl	122 (47)	119 (60)	120 (56)	120 (39)	0.964

Quantitative variables are presented as mean values (standard deviation) or median values {interquartile range}. BMI: body mass index, WC: waist circumference, T2DM: type 2 diabetes mellitus, LSM: liver stiffness measurement, ALT: alanine aminotransferase, AST: aspartate aminotransferase, ALP: alkaline phosphatase, GGT: gamma glutamyl-transferase, INR: international normalized ratio, LDL: low density lipoprotein (cholesterol), HDL: high density lipoprotein (cholesterol), TG: triglycerides. ^1^ NAFL + NASH patients, ^2^ Cirrhotic patients.

**Table 2:** Parameters of circulating cell-free DNA (cf-DNA) species in patients with non-alcoholic fatty liver disease in relation to their disease severity: non-alcoholic fatty liver (NAFL), non-alcoholic steatohepatitis (NASH) and cirrhosis.

	NAFL (*n* = 10)	NASH (*n* = 23)	No Cirrhosis ^1^ (NAFL + NASH)	Cirrhosis ^2^ (*n* = 16)	*p*-Value (1 vs. 2)
cfDNA, ng/mL	72.9 {95.2}	68.4 {86.9}	72.6 {88.0}	27.9 {40.7}	0.002
RNAse P, GE	83.6 (46.9)	63.8 (36.7)	70.2 (40.6)	39.9 (15.5)	<0.001
Alu115, GE	131.4 (81.8)	104.8 (53.8)	112.9 (63.5)	89.7 (45.4)	0.199
Alu247, GE	39.9 (27.7)	31.1 (16.7)	33.8 (20.6)	29.9 (19.0)	0.525
Alu247/Alu115 ratio	0.29 (0.05)	0.30 (0.05)	0.30 (0.05)	0.31(0.06)	0.297
Mitochondrial DNA, copies	19 {17}	26 {40}	25 {37}	27 {27}	0.725
5′methyl-2-deoxy-cytidine, ng/mL	144.2 (39.9)	145.6 (34.6)	145.1 (35.7)	170.3 (38.9)	0.029

Quantitative variables are presented as mean values (standard deviation) or median values {interquartile range}. GE: genomic equivalents. ^1^ NAFL + NASH patients, ^2^ Cirrhotic patients.

**Table 3:** Multivariable logistic regression analyses for significantly associated cf-DNA parameters with presence of cirrhosis adjusted for clinico-epidemiological patient characteristics.

Parameters Included in the Model	Adjusted Odds Ratio (95% CI)	*p*-Value
1st model Age (per 1 year) T2DM (yes vs. no) Cell-free DNA (per 1 ng/mL)	1.130 (1.024–1.247) 3.512 (0.588–20.965) 0.974 (0.951–0.997)	0.015 0.168 0.026
2nd model Age (per 1 year) T2DM (yes vs. no) RNAse P coding DNA (per 1 GE)	1.140 (1.025–1.269) 5.724 (0.626–52.339) 0.945 (0.896–0.996)	0.0160.1220.035
3rd model Age (per 1 year) T2DM (yes vs. no) 5-methyl-2′-deoxycytidine (per 1 ng/mL)	1.142 (1.039–1.256) 2.727 (0.498–14.916) 1.014 (0.994–1.035)	0.0060.2470.163

CI: confidence interval, T2DM: type 2 diabetes mellitus, GE: genomic equivalents.

## Data Availability

The data presented in this study are available on request from the corresponding author.
